# Beneath the surface: non-target effects of multiple pesticides on the soil microbiome in organic and conventional agricultural European fields

**DOI:** 10.1007/s11356-026-38127-7

**Published:** 2026-08-13

**Authors:** Dennis Knuth, Philipp Mäder, Jos Boekhorst, Christian Poll, Ellen Kandeler, Abdallah Alaoui, Igor Pasković, Marija Polić Pasković, Isabelle Baldi, Mathilde Bureau, Francisco Alcon, Josefa Contreras, Matjaž Glavan, Nelson Abrantes, Isabel Campos, Trine Norgaard, Esperanza Huerta Lwanga, Violette Geissen, Paula Harkes

**Affiliations:** 1 Soil Physics and Land Management Group, Wageningen University and Research, Droevendaalsesteeg 3, 6708 PB Wageningen, the Netherlands; 2 Department of Soil Biology, Institute of Soil Science and Land Evaluation, University of Hohenheim, Emil-Wolff-Str. 27, 70593 Stuttgart, Germany; 3 Host-Microbe Interactomics, Wageningen University and Research, De Elst 1, 6708 WD Wageningen, the Netherlands; 4 Institute of Geography, University of Bern, Hallerstrasse 12, 3012 Bern, Switzerland; 5 Department of Agriculture and Nutrition, Institute of Agriculture and Tourism, K. Huguesa 8, 52440 Poreč, Croatia; 6 University Bordeaux, INSERM, BPH, U1219, 146 Rue Léo Saignat, 33076 Bordeaux, France; 7 Escuela Técnica Superior de Ingeniería Agronómica, Universidad Politécnica de Cartagena, Paseo Alfonso XIII, 48, 30203 Cartagena, Spain; 8 Agronomy Department, Biotechnical Faculty, University of Ljubljana, Jamnikarjeva 101, 1000 Ljubljana, Slovenia; 9 CESAM and Department of Biology, University of Aveiro, 3810-193 Aveiro, Portugal; 10 CESAM and Department of Environment and Planning, University of Aveiro, 3810-193 Aveiro, Portugal; 11 Department of Agroecology, Aarhus University, Blichers Allé 20, 8830 Tjele, Denmark; 12 Environmental Science Group, Wageningen University and Research, Droevendaalsesteeg 3, 6708 PB Wageningen, the Netherlands

**Keywords:** SPRINT, Agricultural soils, Pesticides, Microbial community composition, Soil enzyme activity, Phospholipid fatty acids

## Abstract

Previous studies have shown that diverse cocktails of pesticide mixtures are omnipresent in agricultural soils yet miss a clear link to the effects on the soil microbiome. In this study, we linked the occurrence of pesticides in conventional and organic agricultural soils of the SPRINT (Sustainable plant protection transition) projects’ Case Study Sites to the composition and function of soil microbial communities. Metagenomic sequencing, phospholipid fatty acids analysis and enzyme activity measurements were used to characterize the soil microbiome and effects of site-specific parameters such as pH and SOC, and pesticide residues. Differences in the soil microbiome were strongly influenced by the geographic origin of the samples, with the pH value as dominant driver. Against our hypothesis, effects of the investigated management systems were limited, yet significant at the European level. Notably, an association between some pesticides could still be observed after accounting for the variation explained by the environmental factors. Among these, especially fungicides, with modes of action that aim to interfere with processes in microorganisms, seemed to affect the soil microbiome. This might occur either by directly affecting these processes in non-target organisms or by changing co-dependencies between fungi and bacteria. Next to fungicides, aminomethylphosphonic acid showed a significant effect on the soil microbial composition and an interactive, possibly synergistic effect with the persistent pesticide hexachlorobenzene. The latter raises concerns about a possible interaction of recently applied pesticides and persistent “legacy” pesticides. This work highlights that even though environmental parameters can overshadow the effects of pesticides, especially the identity of the pesticides present, can have an influence on the soil microbiome.

**Supplementary Information:**

The online version contains supplementary material available at 10.1007/s11356-026-38127-7.

## Introduction

Current agricultural practices rely on synthetic inputs such as fertilisers and pesticides to ensure crop quality and quantity. In the second half of the twentieth century, concerns about human and environmental health through the application of synthetic pesticides increased. Pesticide use, in particular, has been the subject of extensive research and policy work in recent decades. Nevertheless, the estimated amount of pesticides used worldwide has more than doubled in the last 30 years, from 1.8 to 3.7 Mt a^−1^ (FAO 2024). In the European Union (EU), 423 active substances are authorised, resulting in an even larger number of approved plant protection products (PPPs) (EC 2021). In Germany alone, 1765 products with up to four different active substances are approved for commercial use (BVL 2025), 2561 in the Netherlands (ctgb 2025), 2722 in the Czech Republic (MZE 2025), and 708 in Spain (MAPA 2025). It has long been demonstrated that the presence of multiple pesticide residues is the rule rather than the exception in agricultural soils, including legacy pesticides such as DDT and atrazine that have been used decades ago and are banned today (Riedo et al. 2021; Knuth et al. 2024). While considering the individual impact of active substances, most assessments do not consider the combined impact of the active substances that are present and applied in PPP mixtures. Furthermore, the effects, currently applied substances could have combined with residues already present in the environment, are neglected. Thereby, the real effects of environmental mixtures and agricultural mixtures are yet unknown (Panico et al. 2022).

These mixtures could impact non-target organisms such as soil microorganisms, which are the main inhabitants of the agricultural soil and, therefore, among the first groups of organisms affected by pesticides after their application. They play a major role in ecosystem functioning, such as litter decomposition (Helweg et al. 1998) and nutrient cycling, and are also responsible for the degradation of contaminants, including pesticides (Jacobsen et al. 2008). The effects of pesticides on soil microorganisms vary; some microorganisms can utilise pesticides for energy and nutrients, while others are subject to their toxic effects, leading to shifts in the microbial community (Wu et al. 2014). Those toxic effects depend on the mode of action of the pesticide (Zabaloy et al. 2012) and the amount and frequency of application (Zhang et al. 2016). Additionally, the number of pesticides present can affect the toxicity, as has been shown by interactive effects of multiple pesticides (Meidl et al. 2024; Ni et al. 2025). Those effects can contribute to shaping the soil microbiome by altering microbial diversity, function and abundance (Lo 2010; Sim et al. 2022; Lv et al. 2025).

The interactions of pesticides with soil microorganisms can further be influenced by biotic and abiotic variables. Differences in e.g., soil type and soil properties, temperature or nutrient input can determine the extent of the effects by altering pesticide degradation kinetics or microbial activity (Fierer and Jackson 2006; Siedt et al. 2021). Different environments can also influence the effects that pesticides have on soil microorganisms. As such, Jones and Ananyeva (2001) found different effects of metalaxyl when comparing pine forest, pasture and arable soil, whereas Fierer and Jackson (2006) identified the pH value as a major driver of bacterial diversity between sites. Soil management, such as fertiliser application, wheat straw incorporation and natural regeneration, were found to further affect bacterial diversity during different seasons (Guo et al. 2022). Consequently, soil management can shape the microbial community, as was observed by an increased abundance in organic agriculture when compared to conventional farming (Harkes et al. 2019). In organic farming, the use of pesticides is strongly regulated, yet as was observed by e.g. Froger et al. (2023), residues are still present in these soils. Their persistence in soil could explain their presence, so-called legacy compounds or carryover from neighbouring conventional farms, via e.g. runoff or spray drift. Additionally, especially in organic viniculture, copper-based fungicides are widely used. Copper contamination has previously been shown to affect microbial community structure and ecosystem services (Shaw et al. 2020; Wang et al. 2007). For these reasons, it is important to investigate the differences in soil microbiomes between conventional and organic farming systems in the context of pesticide application.

In this study, we investigated the effects of multiple pesticides on the soil microbial community in organic and conventional fields in ten different European countries, taking the main European cropping systems under consideration (Knuth et al. 2024). We used metagenomic sequencing approaches as well as phospholipid fatty acid analysis (PLFA) and enzyme activity to visualise changes in the microbial community structures and their activity. We aimed to (1) investigate the microbial composition across countries, as understanding microbial composition on a broad geographical scale allows us to identify overarching patterns and regional differences in microbial diversity, which may be driven by soil properties, climate, or management practices, (2) analyse whether the management system affects the microbial composition and function within countries, and (3) observe whether these effects are related to pesticides with a particular mode of action. We hypothesised that there would be a clear difference in the microbial data between organic and conventional systems, as pesticide pressure should be higher in conventional systems. We also contemplated that microbial diversity would be higher in organic systems due to reduced pesticide input. Finally, we conjectured that modes of action that aim to disrupt processes also occurring in microorganisms would have a greater impact on the microbial composition than pesticides that aim to disrupt weed growth or kill insects.

## Materials and methods

### Soil sampling

During the 2021 growing season, soil samples were collected after 50–80% of the anticipated pesticide treatments had been applied in each country. Detailed information on the sampling approach and the case study sites (the CSSs) has been published by Silva et al. (2021). In brief, 200 soil samples were taken in ten European countries, where approximately 20 sites, consisting of half organic farming and half conventional farming, were chosen based on the same crop type per country. The samples were taken in the region of Istria in Croatia (CSS Croatia, olive trees), all over the Czech Republic (CSS Czech Republic, oil seeds), the central zone of Denmark (CSS Denmark, wheat), Bordeaux region in France (CSS France, vine), Po region Italy (CSS Italy, orchards), the Friesland and Groningen regions of the Netherlands (CSS Netherlands, seed potatoes), Bairrada region in Portugal (CSS Portugal, vine), central zone of Slovenia (CSS Slovenia, maize), region of Cartagena in Spain (CSS Spain, vegetables), and the central zone of Switzerland (CSS Switzerland, orchards). They are therefore not representative of the whole country but more of the smaller regions. The soil sampling depth was 0–5 cm or 0–20 cm for permanent and tilled arable land, respectively. Per field, a composite sample of five randomly distributed samples was taken and stored frozen at − 20°C. For pesticide analysis and microbial analysis, one subsample each of the composite sample was stored. The analysed soil physical properties and other characteristics of the CSSs can be found in Table S1. The analysis of 45 insecticides, 50 herbicides, 57 fungicides, 39 metabolites, and the synergist piperonyl butoxide was previously described by Knuth et al. (2024). Additionally, Cu content of the soils was taken into consideration as Cu-sulphates are the most applied fungicides in organic farming systems. The concentrations of the individual substances in the individual samples are listed in Table S1.

### Difference in pesticide composition

Knuth et al. (2024) found in their dataset mixtures from up to 12 different pesticides in organic and up to 21 different pesticides in conventional samples. Leading to 151 different pesticide mixtures, and mixtures in 96% of conventional and 79% of organic fields. They found significant differences in pesticide numbers in seven out of ten countries and in concentrations in five out of ten countries. The maximum sum concentration of pesticide residues was 28.7 mg kg^−1^, and the median sum concentration was 98.2 μg kg^−1^, in organic samples 31 μg kg^−1^ and in conventional samples, 250.1 μg kg^−1^. The analytical techniques are described by Knuth et al. (2024).

### Copper extraction

Bioavailable copper was extracted using a method based on the NEN 5704 (NEN 1996). Soil samples were dried at 40 °C and subsequently extracted at 20 °C with 0.01 mol l^−1^ CaCl_2_ (1:10 ratio, w/v). Samples were shaken for two hours, pH was measured in the settling solution and heavy metals were analysed by ICP-OES.

Total copper was extracted using an AquaRegia extraction based on NEN 6961 (NEN 2023). Air-dried and colloid-grinded soil samples were moistened with AquaRegia (1:16, w/v) and incubated for several hours. Subsequently, samples were placed in a rendering apparatus. After rendering, the samples were allowed to cool down and afterwards replenished and mixed with demineralised water. After settling of the particles, the extract was filtered and analysed with ICP-OES.

### DNA isolation

For the total DNA extraction, 1 g of subsampled soil was used. A Vortex-Genie 2 and silicon carbide powder were used for the disaggregation of soils and cell membranes. Ammonium acetate and ammonium aluminium sulphate dodecahydrate were used for the precipitation of proteins and humic substances, respectively. A binding solution was added and DNA was purified on CommaPrep® RP20 RNA extraction columns. DNA quality and quantity were evaluated by Nanodrop and Qubit. Eluates were stored at − 20°C. The detailed extraction protocol can be found in the supplementary material.

### Sequencing and bioinformatics

Metagenomic sequencing was performed by BaseClear B.V. (Leiden, The Netherlands) using the Illumina MiSeq platform. Compositional profiling of soil metagenomics was done with Kraken2 (Wood et al. 2019) and Bracken (Lu et al. 2017) with the database “2_pluspfp_20220607” (Standard plus protozoa, fungi and plant) as downloaded from https://benlangmead.github.io/aws-indexes/k2. The *name2taxid* function of the *taxizedb* package was used to recreate the taxonomic table from the Bracken output for further downstream analysis in R.

### Phospholipid fatty acid analysis

The abundance of major microbial groups was determined according to Frostegård et al. (1991). Briefly, 4 g field moist soil was extracted with chloroform, methanol and citrate buffer (pH 4) (1:2:0.8 v/v/v) (Bligh and Dyer 1959). Lipids were fractionated using silica acid SPE cartridges (Bond Elut SI, 500 mg, 3 ml, Agilent Technologies Inc, Santa Clara, CA, USA) and the fraction containing the phospholipid fatty acids was collected. Using an alkaline methanolysis (Kramer et al. 2012), PLFAs were transformed into fatty acid methyl esters (FAMEs) and measured on a gas chromatograph (GC) (AutoSystem XL, Perkin Elmer Inc., USA) equipped with an HP-5 capillary column and flame ionisation detector with helium as carrier gas. Following Ruess and Chamberlain (2010), we classified FAMEs i15:0, a15:0, i16:0, and i17:0 as gram-positive bacteria, cy17:0 and cy19:0 as gram-negative bacteria, and the sum of gram-positive and gram-negative markers together with 16:1ω7 as the total bacterial FAMEs (Ruess and Chamberlain 2010). The FAME 18:2ω6,9 was used as a fungal biomarker. Ratios of the fungal FAME and the total bacterial FAMEs, as well as the ratio between gram-positive and gram-negative bacterial FAMEs were calculated.

### Activities of enzymes involved in C-, N- and P-cycling

The activity of four enzymes involved in the C-, N-, and P-cycling was determined according to Marx et al. (2001) using fluorogenic 4-methylumbelliferone (MUF) substrates for the enzymes β-glucosidase (EC 3.2.1.21), β-xylosidase (EC 3.2.1.37), N-acetyl-β-glucosaminidase (EC 3.2.1.14) and acid phosphatase (EC 3.1.3.2). According to Poll et al. (2006), substrates and MES buffer were prepared. After mixing one gram of soil with 50 ml of autoclaved H_2_O_deion._, it was dispersed with an ultrasonic disaggregator (50 J s^−1^) for 2 min. While stirring on a magnetic stir plate, 50 µl of this suspension was transferred into 96-well microplates (PP F black 96 well, Greiner Bio-one GmbH, Germany) and filled with 50 µl of MES buffer and 100 µl of the substrate solution. Standards were created using 50 µl of the soil suspension with 0, 100, 200, 500, 800 or 1200 pmol/well MUF, resulting in a final volume of 200 µl. After pre-incubation for 30 min in the dark at 30 °C, the plates were exposed to a wavelength of 360 nm using a microplate fluorescence reader (Bio-Tek Instruments Inc., FLX 800, Germany) and measured at a fluorescence intensity of 460 nm. Temporal resolution was achieved by measuring the fluorescence after 0, 30, 60, 120 and 180 min. Enzyme activities were linearly correlated to the fluorescence and calculated based on the standards in nmol g^−1^ h^−1^.

### Data analysis

Data analysis was conducted in R using mainly the packages *phyloseq*, *microViz* and *stats*. As *Homo sapiens* was one of the identified species, its count was removed from the dataset. Compositional data was rarefied with the *rarefy_even_depth* function (set.seed (123) was used to initialise repeatable random subsampling) of the *phyloseq* package (McMurdie and Holmes 2013), using the lowest read for standard sequencing depth. This was done to account for different sequencing depths (Ranging from 6.5 million to 33 million paired-end reads, with a median of 12.6 million.). The α-diversities and PCA/RDA analysis were conducted in *phyloseq*, and PERMANOVAs were calculated with *microViz* package (Barnett et al. 2021). To fit linear models the *stats* package was used (R Core Team 2013). For significantly differing biological parameters (α-diversity, enzyme activity and PLFA content) between the management systems within a country, in an exploratory approach, linear models for the explanation of these parameters were generated. Linear models were fitted with the significantly differing biological parameters as dependent and SOC, pH, water content, clay content, total copper, AMPA and the sum per different chemical classes and types of pesticides as explanatory variables (details can be found in the supplementary material Tables S1 and S2). When one of the sum parameters (type of pesticide or chemical class) was included in the model, it got deleted (for type of pesticides) or replaced by the pesticides it sums up (chemical class, Table S2). Afterwards, the linear model was generated again. Models were fitted as long as AIC difference was ≥ 2. The *p*-values were adjusted for multiple testing with the p.adjust method in R using Benjamini–Hochberg correction (method = “fdr”). Benjamini–Hochberg correction was used to keep the statistical power in the larger number of tests, while reducing the false discovery rate appropriate for a study based on soil monitoring. The *p*-values were adjusted when multiple testing occurred, and the results were used for subsequent analysis. The statistical workflow of the analysis is displayed in Fig. S1.

## Results

### Metagenomic approach

Metagenomic sequencing identified a total of 3563 species, and 3414 species were retained after rarefaction for further analysis. Of these, 3270 were bacteria and 33 fungi, while the rest belonged to other groups, such as eukaryotes, viruses, and archaea. The most abundant phyla were Proteobacteria and Actinomycetota, followed by Euryarcheota, Bacillota, Acidobacteria and Thaumarchaeota. The microbial composition is displayed in Table 1 and is further detailed in Figs. S2 and S3.

**Table 1:** Distribution of the different phyla related to the total microbiome (a) and bacteria only (b) (%) per management system across all soils

All	Proteobacteria	Actinomycetota	Euryarchaeota	Bacillota	Thaumarchaeota
All samples	34.2 ± 11.1	30.9 ± 11.2	18.2 ± 13.4	4.1 ± 3.8	3.8 ± 6.3
Conventional	34.0 ± 11.4	30.8 ± 11.1	17.4 ± 14.0	3.9 ± 3.1	4.4 ± 7.5
Organic	34.5 ± 10.8	31.0 ± 11.3	19.1 ± 12.9	4.4 ± 4.3	3.2 ± 4.7
Bacteria	Proteobacteria	Actinomycetota	Bacillota	Acidobacteria	Planctomycetota
All samples	45.0 ± 13.1	39.9 ± 12.0	5.4 ± 4.8	3.2 ± 3.6	2.7 ± 6.7
Conventional	44.6 ± 13.5	39.7 ± 11.9	5.2 ± 4.2	3.1 ± 3.5	1.5 ± 0.5
Organic	45.4 ± 12.8	40.1 ± 12.2	5.7 ± 5.4	3.3 ± 3.8	1.5 ± 0.6

**Fig. 1:**
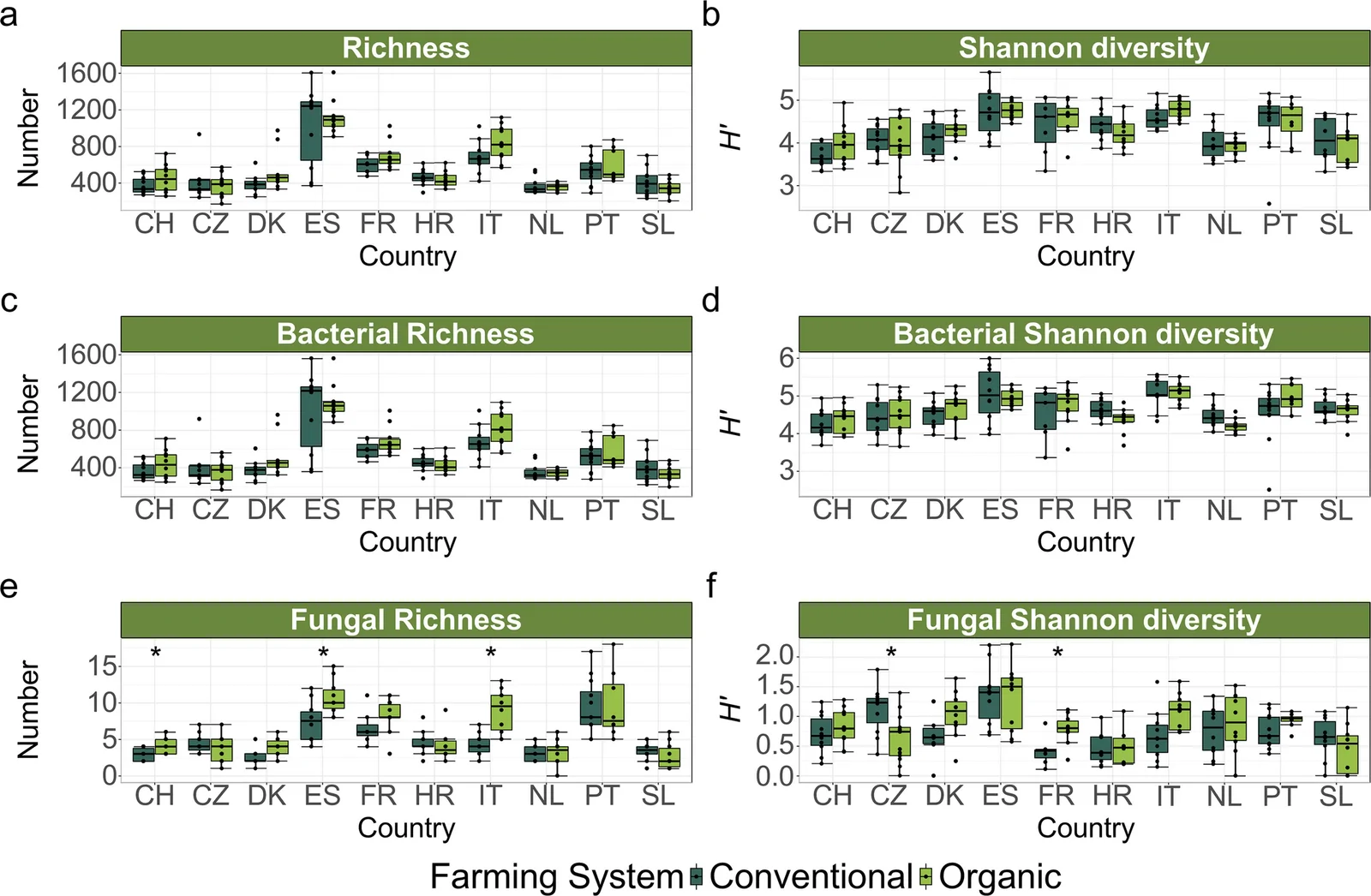
Species richness and Shannon diversity across CSS and management systems. (**a**) and (**b**) for the whole microbial community, (**c**) and (**d**) for only bacteria and (**e**) and (**f**) for fungi only. CSS Switzerland (CH), CSS the Czech Republic (CZ), CSS Denmark (DK), CSS France (FR), CSS Croatia (HR), CSS Italy (IT), CSS the Netherlands (NL), CSS Portugal (PT), CSS Slovenia (SL) and CSS Spain (ES). * *p* < 0.05

No significant differences in α-diversity (Richness or Shannon) were detected between organic and conventional management at the European scale or within individual countries (Fig. 1a and b). However, by dividing the dataset into bacteria and fungi, significant country-specific differences were found: fungal Richness was higher in organic fields in the CSSs Switzerland (*p* = 0.046), Spain (*p* = 0.046), and Italy (*p* = 0.0003) (Fig. 1e). Fungal Shannon diversity was significantly higher in conventional fields in CSS Czech Republic (*p* = 0.042), as well as in organic fields in CSS France (*p* = 0.042) (Fig. 1f). These differences did not follow a consistent pattern across countries.

**Fig. 2:**
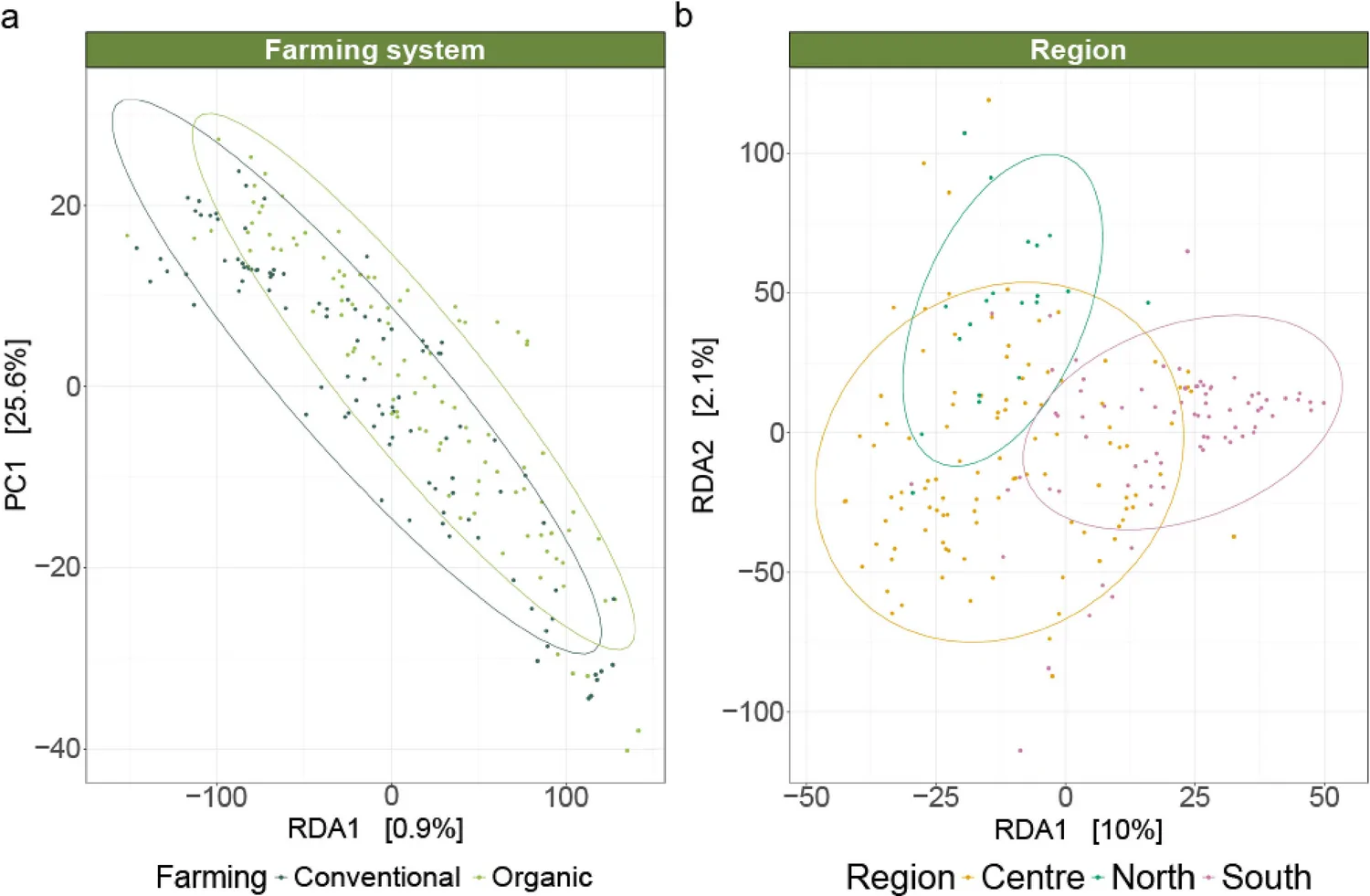
(**a**) Redundancy analysis of all samples with the management system as the explanatory variable. (**b**) Redundancy analysis (RDA) of all samples with the region as an explanatory variable. Ellipsis mark samples from central Europe (yellow), North Europe (green), and South Europe (purple)

To investigate the effect of the management system on the microbial composition, redundancy analyses were performed. This showed that management had only a minor influence on microbial community composition at the European scale. When used as the explanatory variable, the management system accounted for 0.9% of the variation along RDA1, showing a large overlap between the organic and conventional fields. In contrast, geographical factors explained much more of the community structure: ‘region’ accounted for 10% of the variation, clearly separating northern and southern European countries (Fig. 2a and b), but both overlapping with the central European samples. Using ‘country’ as the explanatory variable, 13% of microbial community variation was explained (Fig. S4).

When correcting for the country effect, the influence of management remained small, explaining only 1.1% of the variation, although still statistically significant. PERMANOVA results showed the same pattern: management system explained 0.92% of the variation (*p* = 0.047), whereas region (16.8%, *p* = 0.001) and country (43.2%, *p* = 0.001) were the dominant drivers. After correcting for country, the effect of management remained significant but small (0.92%, *p* = 0.002).

To determine whether crop type influenced these patterns, we compared case study sites growing the same crops. Even within crop groups (the CSSs France and Portugal, grapes and the CSSs Spain and Italy, vegetables), the country effects remained significant (8.1% (*p* = 0.003)) and (28.9% (*p* = 0.001)), respectively, on the microbial composition (RDA in Fig. S5).

**Fig. 3:**
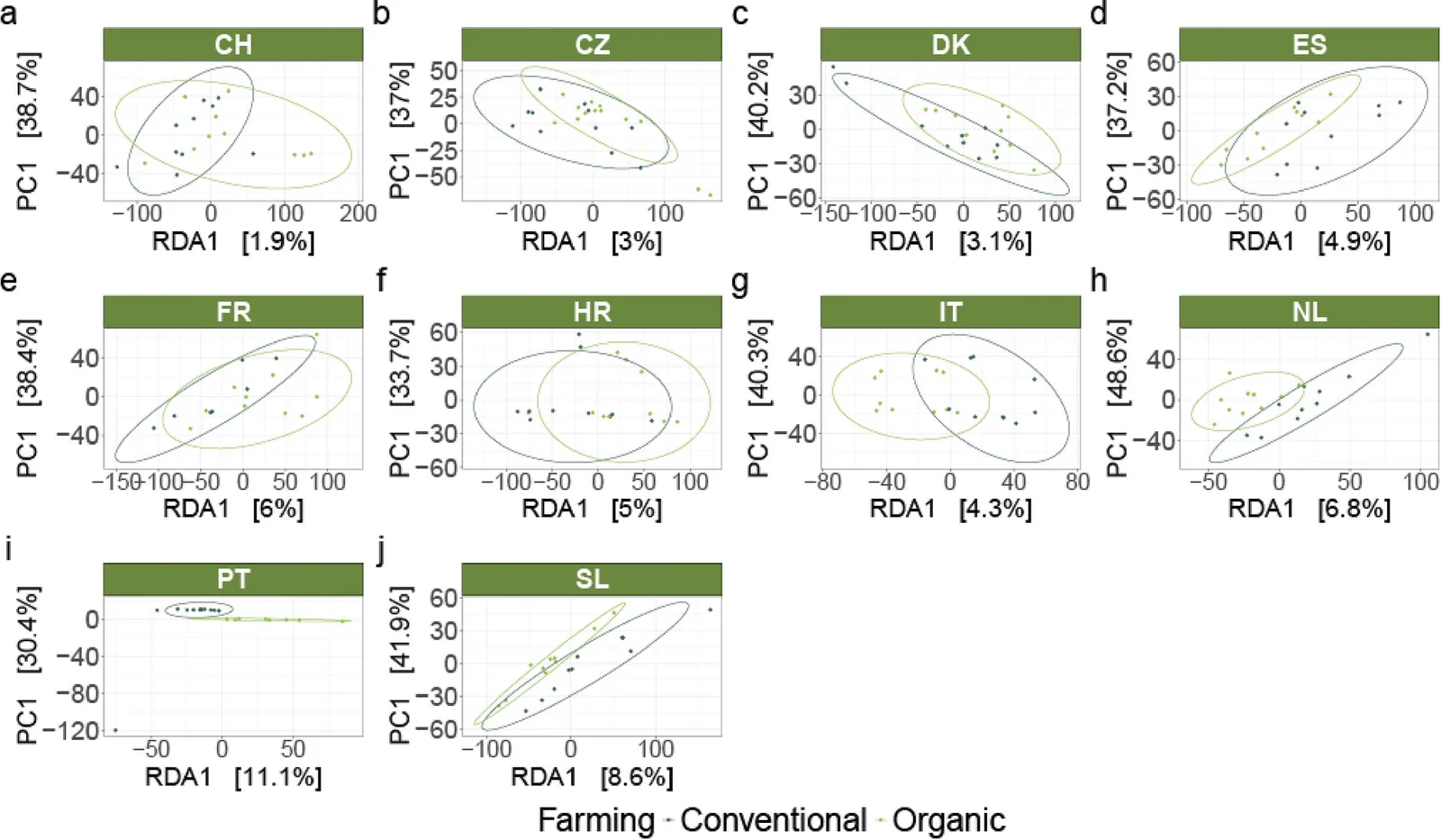
Redundancy analyses per country (**a**) CSS Switzerland (CH), (**b**) CSS the Czech Republic (CZ), (**c**) CSS Denmark (DK), (**d**) CSS France (FR), (**e**) CSS Croatia (HR), (**f**) CSS Italy (IT), (**g**) CSS the Netherlands (NL), (**h**) CSS Portugal (PT), (**i**) CSS Slovenia (SL) and (**j**) CSS Spain (ES) using the management system as the explanatory variable

At the country level, RDA showed that the proportion of variation explained along RDA1 varied widely among case study sites. Across all countries, RDA1 accounted for 1.9–11.1% of the variation in microbial composition (Fig. 3), while the management system explained 2.9–11.5%, with a significant effect detected only in Portugal (Table S3). When analysing bacterial communities separately, RDA1 explained 1.7% and 18.5% of the variation (Fig. S6), showing clear separations between management systems in the CSSs of France, Croatia, the Netherlands, Portugal, and Spain. The management system accounted for 2.8–10.8% of community variation and was significant in the CSSs of Spain, Portugal, Italy, and the Netherlands. For fungi, RDA1 explains 1.8–17.6%, and management explained 2.1–17.6% with significant effects in the CSSs Denmark, Italy, and the Netherlands.

Using the number of pesticides as the explanatory variable, RDA1 accounted for 0.9%–11.7% of variation in microbial composition (Fig. S7). Pesticide number itself explained 1.8%–14.6% of variation in microbial composition and showed significant effects in the CSSs Netherlands and France (Table S4). After correcting for management, RDA1 explained 3.6 and 12.1% of community variation (Fig. S8), although PERMANOVA detected a significant effect only in CSS France (Table S5).

To identify the environmental and chemical factors shaping the microbial community, an RDA and PERMANOVA were performed using all pesticides present in 20 or more of the analysed samples, total and bioavailable copper, the physical soil parameters (SOC, water content, texture), as well as the management system and country. Country and pH were significant explanatory variables for the whole community, bacteria, and fungi. Texture, AMPA, azoxystrobin, metolachlor OA and difenoconazole were significant in the whole community and the bacteria, whereas SOC was only significant in bacteria and fungi, but not in the whole community. Metalaxyl-M and total copper were significant for the whole community only (Table 2).

**Table 2:** PERMANOVA results, *p*-values of pesticides with an occurrence in 20 or more fields and other abiotic variables for the whole community and for bacteria and fungi only. Pesticide types: *F*, Fungicide; *H*, Herbicide; *I*, Insecticide and *M*, Metabolite. *P*-values were fdr adjusted

	Typ	Count	All					Bacteria					Fungi				
			R2	Pr(> F)		P adj		R2	Pr(> F)		P adj		R2	Pr(> F)		P adj	
Country			0.1854	0.001	***	0.013	**	0.1944	0.001	***	0.013	*	0.1631	0.001	***	0.026	*
Farming			0.0036	0.13		0.267		0.0028	0.233		0.401		0.0057	0.059		0.322	
Texture			0.0359	0.016	*	0.111		0.0318	0.030	*	0.082		0.0303	0.554		0.686	
SOC			0.0036	0.134		0.270		0.0048	0.023	*	0.082		0.0092	0.010	**	0.087	
pH			0.0398	0.001	***	0.013	**	0.0516	0.001	***	0.013	*	0.0091	0.007	**	0.052	
Water content			0.0036	0.113		0.270		0.0038	0.076		0.164		0.0024	0.495		0.686	
p,p'-DDE	M	180	0.0030	0.270		0.354		0.0019	0.646		0.675		0.0032	0.342		0.633	
AMPA	M	74	0.0053	0.025	*	0.127		0.0051	0.013	*	0.082		0.0046	0.132		0.442	
HCB	F	81	0.0027	0.358		0.399		0.0022	0.431		0.524		0.0034	0.308		0.633	
Chlorpyrifos	I	69	0.0014	0.894		0.892		0.0014	0.867		0.856		0.0031	0.370		0.633	
Glyphosate	H	42	0.0029	0.26		0.367		0.0032	0.150		0.273		0.0017	0.758		0.814	
p,p'-DDT	I	51	0.0036	0.126		0.267		0.0023	0.445		0.519		0.0040	0.192		0.494	
Boscalid	F	47	0.0032	0.198		0.312		0.0030	0.157		0.286		0.0015	0.831		0.858	
o,p'-DDT	I	36	0.0033	0.178		0.270		0.0023	0.443		0.519		0.0025	0.529		0.686	
p,p'-DDD	M	33	0.0023	0.490		0.582		0.0016	0.753		0.772		0.0017	0.780		0.814	
Azoxystrobin	F	29	0.0049	0.032	*	0.111		0.0047	0.019	*	0.082		0.0024	0.512		0.686	
Tebuconazole	F	30	0.0023	0.556		0.583		0.0019	0.642		0.675		0.0022	0.596		0.736	
λ-Cyhalothrin	I	26	0.0021	0.587		0.591		0.0023	0.409		0.519		0.0038	0.217		0.494	
Metalaxyl-M	F	28	0.0048	0.021	*	0.127		0.0038	0.063		0.164		0.0029	0.376		0.645	
Epoxiconazole	F	20	0.0040	0.087		0.218		0.0027	0.237		0.410		0.0044	0.148		0.442	
Difenoconazole	F	23	0.0045	0.047	*	0.144		0.0050	0.023	*	0.082		0.0055	0.051		0.322	
Fluopyram	F	23	0.0030	0.243		0.354		0.0029	0.217		0.333		0.0051	0.094		0.381	
Fluopicolide	F	22	0.0028	0.317		0.378		0.0036	0.083		0.198		0.0018	0.702		0.814	
Metolachlor OA	M	20	0.0050	0.013	*	0.127		0.0040	0.049	*	0.164		0.0030	0.357		0.633	
Cu (total)		201	0.0049	0.027	*	0.270		0.0035	0.100		0.220		0.0024	0.544		0.454	
Cu (Ca^2+^)		53	0.0035	0.124		0.127		0.0034	0.129		0.197		0.0042	0.169		0.686	
–-																	

Signif codes: ‘***’ *p* < 0.001 ‘**’ *p* < 0.01 ‘*’*p* < 0.05

When analysing the most frequently detected synthetic pesticides (*n* ≥ 20) individually and correcting for country, several compounds showed significant associations with microbial community composition. Corrected for country, six commonly detected pesticides (metalaxyl-M, AMPA, metolachlor OA, boscalid, difenoconazole, and glyphosate) significantly affected microbial community composition. AMPA and difenoconazole affected bacteria, while AMPA was the only compound affecting fungi: all results are displayed in Table S7.

To evaluate potential combined effects of multiple pesticides on the microbial composition, we used RDAs and PERMANOVAs for the three most commonly detected pesticide residues together with the significant pesticides from the analysis described above. HCB and AMPA were significant drivers of community structure for the whole microbiome in all combinations; additionally, difenoconazole and metolachlor OA showed significant effects (Table S8). Looking only at the bacterial community, AMPA was significant in all combinations where it was present, while HCB became significant only when AMPA was present. Additionally, difenoconazole and metalaxyl-M were significant (Table S9). On the fungal community, AMPA showed a significant effect in every combination in which it was present (Table S10).

### Phospholipid fatty acid analysis and enzymatic activity

**Fig. 4:**
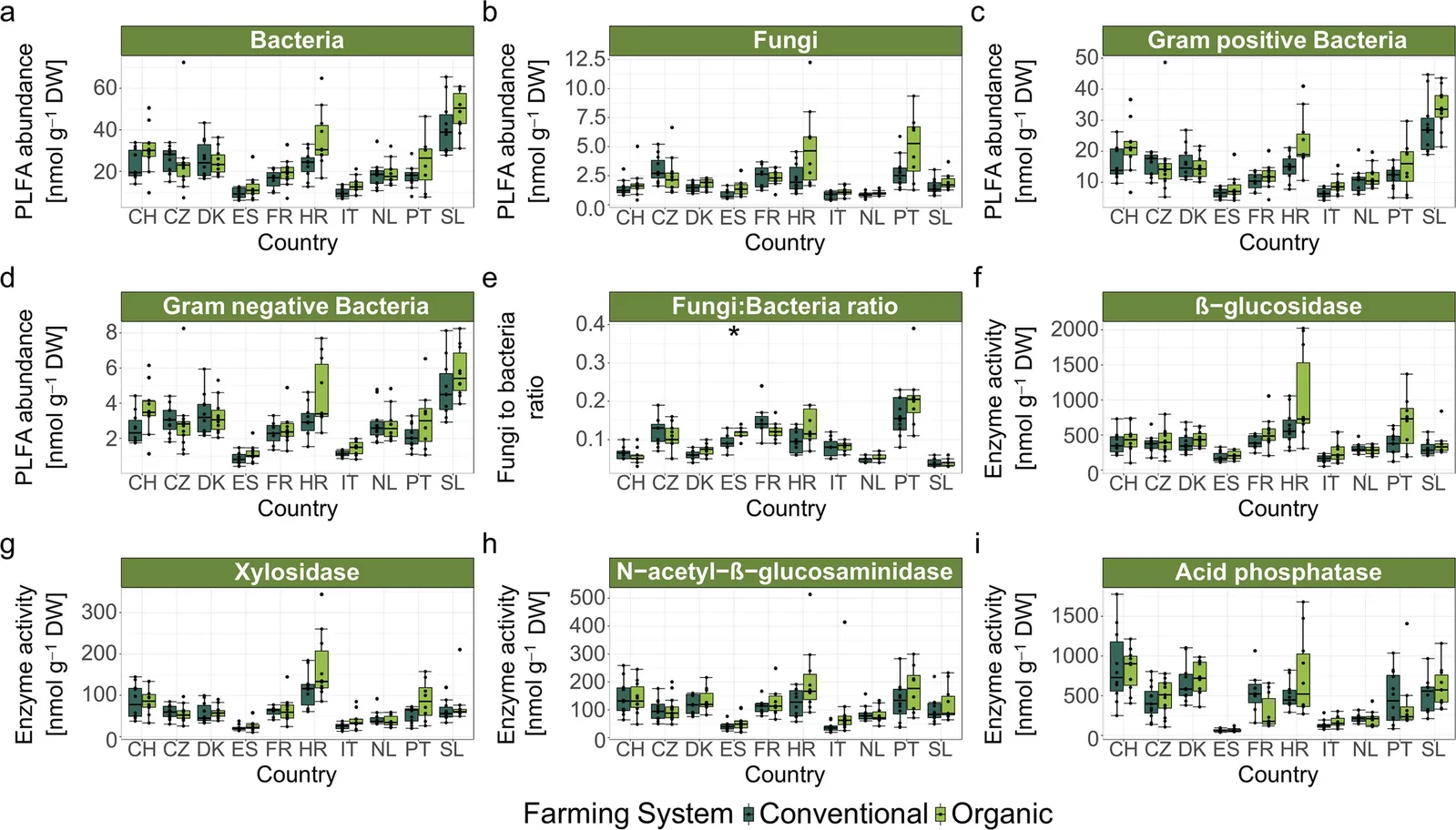
Boxplots and significance of different measured biological parameters (**a**) bacterial PLFA, (**b**) fungal PLFA, (**c**) PLFA of gram-positive bacteria, (**d**) PLFA of gram-negative bacteria, (**e**) ratio of fungal and bacterial PLFA, (**f**) beta-Glucosidase, (**g**) Xylosidase, (**h**) Chitinase and (**i**) Acid-Phosphatase). CSS Switzerland (CH), CSS the Czech Republic (CZ), CSS Denmark (DK), CSS France (FR), CSS Croatia (HR), CSS Italy (IT), CSS the Netherlands (NL), CSS Portugal (PT), CSS Slovenia (SL) and CSS Spain (ES). In (nmol g^−1^DW). * *p* < 0.05

PLFA profiles showed few differences between management systems. A significant difference in the fungal-to-bacterial ratio was detected only in CSS Spain (Fig. 4a–e). Likewise, the activities of the four measured enzymes (β-glucosidase, β-xylosidase, N-acetyl-β-glucosaminidase and acid phosphatase) did not differ significantly between the management systems in any country (Fig. 4f–i).

**Fig. 5:**
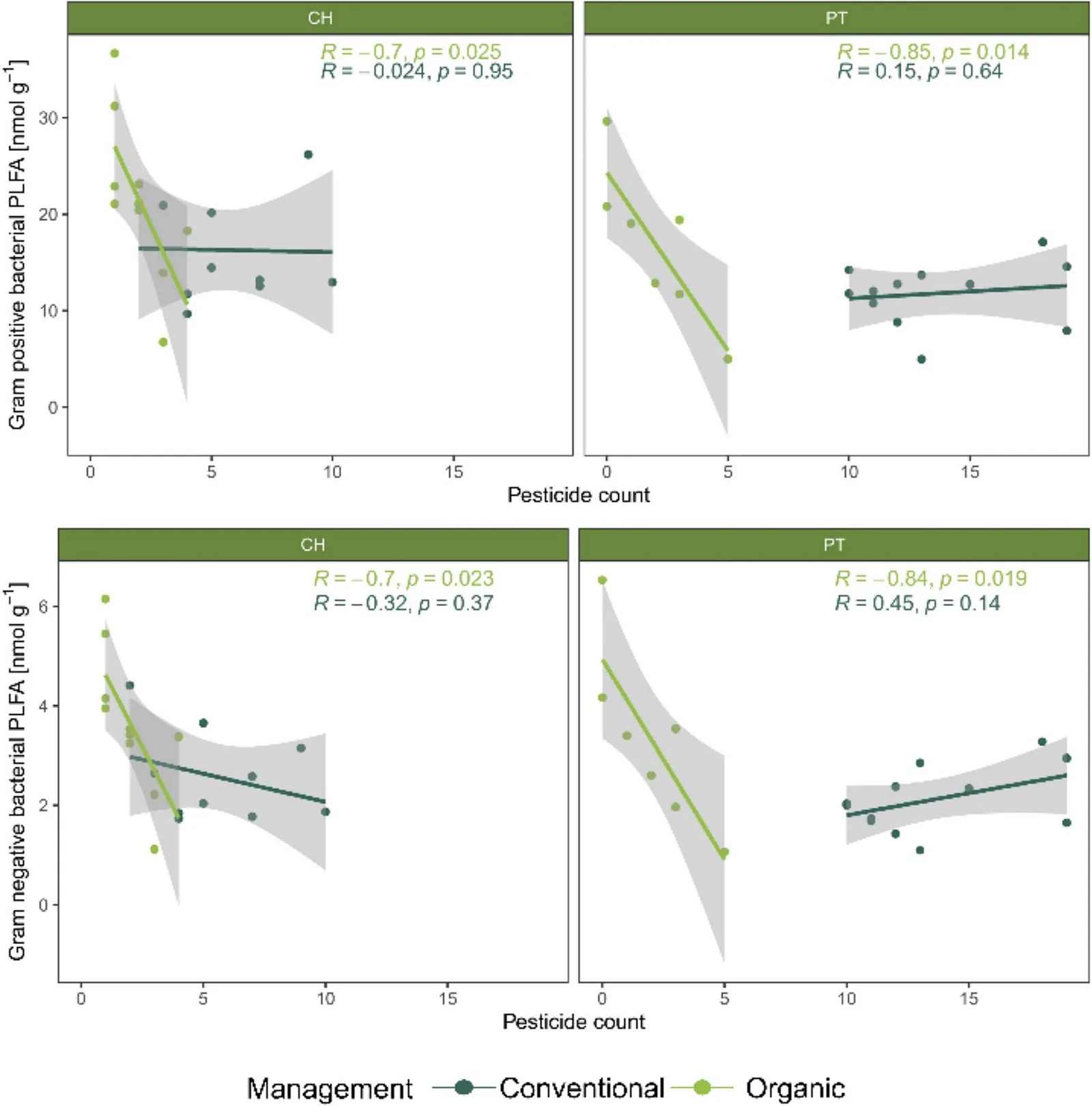
Correlation of detected pesticide number and bacterial PLFA concentration (nmol g^−1^) for in the upper segment gram positive and in the bottom segment gram negative for CSS Switzerland (CH) and CSS Portugal (PT) divided by management system

To explore whether pesticide exposure influenced microbial groups or activity, we correlated the number of detected pesticides with the PLFA-based variables and the enzyme activity measured. In the CSSs Switzerland and Portugal, both gram-positive as well as gram-negative bacteria in organic fields declined with increasing pesticide number, while it was relatively stable in conventional fields (Fig. 5). In general, microbial communities in organic systems appeared more sensitive to increasing pesticide counts than those in conventional systems (Fig. S9). Across countries, most microbial parameters showed a slight decrease with higher pesticide numbers (Fig. S10).

### Combined approach of linear models and metagenomics

At the European scale, multivariate analyses indicated that pesticide-related variables contributed to variation in soil microbial community composition despite strong environmental and regional effects. In an exploratory approach to identify regional factors associated with the biological differences observed between the two management systems within countries, we fitted linear models for each country that exhibited significant differences between the management systems. These include SOC, pH, water content, clay content, total copper concentration, and the molecular concentration of various pesticide classes. Linear models incorporating pesticides are presented in Table 3, all of the presented models were significant (for all statistics, see Table S6). The pesticides featured in these linear models included five fungicides (HCB, boscalid, fluopicolide, trifloxystrobin, and propamocarb (HCl)), and three metabolites (AMPA, p,p'-DDE, o,p'-DDT).

**Table 3:** Linear models for measured biological variables with significant differences between the management systems in a specific country. Including adjusted *R*^2^ and *p*-value of the models. Only linear models including pesticides are displayed

Country	Variable	Model	Adjusted R-squared	*p*-value
CSS Switzerland	Fungal richness	~ HCB + Cu (tot) + p,p’-DDE + AMPA + clay + SOC + trifloxystrobin	0.618	0.001
CSS Italy	Fungal richness	~ pH + propamocarb (HCl) + boscalid	0.387	0.012
CSS France	Fungal Shannon diversity	~ fluopicolide + AMPA + Cu (tot) + trifloxystrobin + p,p’-DDT + water content + SOC + pH	0.874	0.001

In Switzerland, fungal richness was significantly associated with HCB, total copper and p,p′-DDE. In Italy, fungal richness was influenced by propamocarb (HCl) and by pH. Fungal Shannon diversity was significantly associated with water content, AMPA, SOC, fluopicolide and total copper (Table 3).

Subsequently, we used the variables retained in the linear models as explanatory variables in the country-specific RDAs. While a total of 9 pesticide residues were included in the linear models, only p,p'-DDE, as well as total copper content, and pH, showed significant effects on the microbial composition of the corresponding the CSSs (Table 4). In CSS Switzerland, p,p'-DDE significantly affected the total community while the total copper and SOC explained the variation in the bacterial community. In the CSSs Italy and France, pH affected both the whole and bacterial community and in CSS Italy it also affected the fungal community. In contrast, AMPA and trifloxystrobin, although present in two of the linear models, did not show significance in any RDA.

**Table 4:** Formulas retrieved from the linear models were used for RDAs in specific countries. The percentage of difference in microbial composition was explained by the variables used as an explanatory variables for the complete microbiome, as well as for bacteria and fungi separately. Significant variables and *p*-values for these variables

Country	Formula	% all	% Bacteria	% Fungi	Sig. Variable	*p*-value
CSS Switzerland	~ HCB + Cu (tot) + p,p’-DDE + AMPA + clay + SOC + trifloxystrobin	47.8*	47.8**	35.7	All: p,p'-DDE Bac: Cu (total) p,p'-DDE SOC	0.031 0.035 0.004 0.028
CSS Italy	~ pH + propamocarb (HCl) + boscalid	16.7*	19.4***	34.5**	All: pH Bac: pH Fun: pH	0.028 0.001 0.002
CSS France	~ fluopicolide + AMPA + Cu (tot) + trifloxystrobin + p,p’- DDT + water content + SOC + pH	65.5*	68.5*	53.5	All: pH Bac: pH	0.042 0.017

Significance: ‘***’ *p* < 0.001 ‘**’*p* < 0.01 ‘*’ *p* < 0.05

## Discussion

Previous studies investigating the occurrence of pesticides found that not only single pesticides are present in agricultural soils but that various pesticide mixtures are more the rule than an exception (Riedo et al. 2021; Knuth et al. 2024). While these studies have recorded the widespread occurrence of pesticide mixtures in agricultural soils, their cumulative and interactive effects on non-target soil microbial communities are still poorly understood. By utilising samples from the SPRINT field sampling campaign, and by combining metagenomic, enzymatic, and PLFA analyses with pesticide residue data, our study offers new insights into how management systems and pesticide types influence soil microbiomes. We could show that regional differences in soil properties do not solely govern differences in the soil microbiome between organic and conventional management systems, but also that pesticides play a role in shaping the soil microbiome.

### The soil microbiome of European soils affected by regional factors and management practices

Overall, the dominant phyla in the European soils did not differ and were not affected by regional differences or management systems within the region. Similar dominant phyla including fungal phyla were observed by various other studies (Rachwał et al. 2021; Labouyrie et al. 2023; Górska et al. 2024). However, we identified that soil-specific characteristics, such as pH value, SOC, and texture, shaped the microbial community, which aligns with the main drivers identified in the literature (Wang et al. 2019; Tian et al. 2021; Walder et al. 2022). Nevertheless, management practices including tillage, crop rotation and fertilisation can influence the soil microbiome (Kong et al. 2023; Mo et al. 2024; Zong et al. 2024). In this study, management was represented by organic and conventional management systems, which per regulations, differ at least in their use of synthetic pesticides (Fess and Benedito 2018). We found a moderate but significant effect of management when evaluated on the European level, which could be caused by the high variability of other environmental factors (e.g. SOC, pH) overshadowing the effect. Other authors who were able to show the influence of soil management practices on fungal and bacterial community structure as well as microbial abundance and activity (Hartman et al. 2018; Harkes et al. 2019) investigated on a smaller scale, resulting in less influence of the spatial sample distribution. However, when we assessed the effects of the management on the country level for different microbial groups, significant differences in microbial composition were observed in only five out of ten countries. This suggests that while our first hypothesis remains valid in some cases, its validity is influenced by country-specific factors that can modulate or even mask the influence of management practices.

Fungal α-diversity was higher in organic farms, showing an association with the management system. Contrary, Karanja et al. (2020) observed an increased fungal diversity in conventional farming compared to organic. However, they also stated that this was due to the use of organic and inorganic inputs in conventional farming. We found a strong association of fungal α-diversity and pH. Similarly, it has previously been shown in southwestern China (Liu et al. 2018), northern Europe (Tedersoo et al. 2020) as well as on the global scale (van Galen et al. 2025) that pH is a major driver for soil microbial diversity, especially of fungi. We could argue that the effect of pH can be related to the management system, as long-term conventional farming can acidify soil through the input of mineral fertilisers (Fliessbach et al. 2024). As this was mainly visible for fungi, and not linked to the presence of different pesticides, our second hypothesis—that microbial diversity would be higher in organic systems due to an absence of pesticide input—cannot be confirmed. However, these results show that physicochemical soil parameters are an important aspect that can shape soil microbial and specifically fungal communities. In line with the minor effects of the management systems on the microbial abundance and diversity, the microbial function, as shown by the enzyme activities, did not differ significantly between the management systems. While García-Ruiz et al. (2008) could show overall differences in enzymatic activities between organic and conventional sites, they concluded that this was not possible when comparing paired sites. We suggest that here, due to the minor effects of the management on microbial abundance and diversity, no effects on their activity were observed.

### Link between management systems, pesticide number and microbial abundance and function

Enzymatic activities were not affected by the presence of pesticides, likely due to only transient changes of the enzymatic activities depending on the pesticide identity and concentrations (Niemi et al. 2009; Riah et al. 2014). Such transient changes have been shown recently for the herbicide prosulfocarb, where two enzymes were temporarily stimulated to overcome potential negative effects of the pesticide (Mäder et al. 2025). Another explanation is the functional redundancy in complex microbial communities, where different taxa have the ability to carry out the same functions, leaving them unaffected when the abundance of one taxon decreases (Allison and Martiny 2008). However, we were able to identify a negative correlation between the number of pesticides and the abundance of gram-positive and gram-negative bacteria in two countries. This pattern was only observed in organic fields, while microbial abundance remained stable in conventional fields. One explanation is that microbial communities in conventional systems, exposed to continuous pesticide use, may have developed greater tolerance, resistance or acclimation to pesticide pressure. This would then also indicate that the microbial communities in the organic management systems were more sensitive to the pesticides or consisted of a community with a higher proportion of sensitive microorganisms. The cause of the observed decrease in organic fields remains unclear. We propose, in accordance with Riedo et al. (2022), that in addition to legacy compounds, the occurrences of pesticide residues in organic fields are a result of the transport of pesticides from surrounding fields. We argue that this would result in the exposure of a microbial community more sensitive to pesticide input, and therefore changes to the abundance of microorganisms can occur that are not visible in an adapted microbial community of the conventional management systems.

While this effect was dependent on the management system, a significant association of the microbial community with three fungicides (difenoconazole, metalaxyl-M and copper), and two metabolites of herbicides (AMPA and metolachlor OA) was independent of the management system. This association might be due to the ability or inability of some genera to utilise pesticides as a carbon and energy source (Wang et al. 2020; Guerrero Ramírez et al. 2023). Individual tolerances or resistance against the toxic effects of pesticides (Shahid and Khan 2022) might also be responsible for the observed effects. Considering that copper is used to control pathogenic fungi, our findings of three fungicides suggest that they are stronger associated with shifts in the soil microbiome than e.g., herbicides or insecticides. An explanation might be that fungicides, per definition, directly affect microorganisms. All of the observed fungicides affect a specific mechanism in targeted pathogenic fungi: difenoconazole inhibits ergosterol synthesis (Vanden Bossche et al. 1990) and metalaxyl-M affects the fungal nucleic acid synthesis (Lewis et al. 2016). This, on the one hand, indicates that the mode of action of these fungicides is non-specific for fungi and on the other hand, through an interaction with bacteria, not limited to only fungi. While we demonstrate that the interaction with fungi is likely associated with changes to the processes affected by the fungicides, bacteria were likely affected indirectly. This is in line with our hypothesis that pesticides aiming to disrupt microbial processes can have a larger effect on soil microorganisms. The fungicides might be associated with disruptions of interactions of bacteria and fungi (Pawlowska 2024) by changing competition, opening of ecological niches formerly occupied by fungi, or direct dependencies (de Boer et al. 2005; Wang & Kuzyakov 2024). This is supported by the findings of Meyer et al. (2024) that more than 75% of the effects caused by the fungicide hymexazol were linked to the disruption of microbial interactions. By changing, e.g., the proportions of microbial groups, functions occupied by these groups may also shift, leading to changes in nutrient cycling or decomposition of various substrates (Riedo et al. 2025). This might be additionally interfered with, when the interactions between microbial groups are altered, especially considering the different roles of fungi and bacteria in these processes (de Boer et al. 2005).

Yet, not only fungicides were associated with changes in the soil microbiome in the European soils. AMPA — the metabolite of glyphosate — affected the composition of the microbial community. The observed decrease in fungal diversity in two countries (CSS Switzerland and CSS France) with increasing AMPA concentrations might indicate that less tolerant fungi were reduced in relation to the AMPA concentration. This was also observed by Aslam et al. (2024), who showed that AMPA induced changes to both fungal and bacterial communities. They showed that while some fungi were only present or more abundant in AMPA treatments, others were highly abundant in glyphosate-treated soil but not in AMPA-treated ones, indicating that differences in tolerances caused these shifts. However, since we only observed a significant effect on the fungal Shannon index and species Richness but not fungal PLFA expression, we argue that the effects are caused by a shift in community structure rather than changes to the total fungal abundance.

Still, it should not be disregarded that the presence of AMPA is a direct effect of glyphosate application and its degradation, and all effects of AMPA should be accounted for as such. Therefore, the observed effects of AMPA could be either caused by AMPA itself, or as a consequence of glyphosate effects, directly or during its degradation. glyphosate targets the 5-enolpyruvylshikimate 3-phosphate (EPSP) synthase in the Shikimate pathway of plants, which leads to a disruption of the biosynthesis of aromatic amino acids in the plant (Schönbrunn et al. 2001). The Shikimate pathway is not uniquely found in plants, but also in other organisms, including bacteria and fungi (Kishore and Shah 1988). As AMPA does not affect the EPSP synthase (Blot et al. 2019; Iori et al. 2020), we suggest that this effect is due to the previous presence of glyphosate or secondary effects, e.g., the absence of weed species as interaction partners.

Furthermore, we found that the presence of AMPA could enhance the effect of the fungicide HCB on the bacterial community composition, suggesting the possibility of a synergistic effect between the two pesticide residues. HCB had the second-highest detection rate of all analysed pesticide residues (Knuth et al. 2024), is known for its high persistence in soil systems and its possible occurrence as metabolite of other substances (Lewis et al. 2016). The potential interaction of AMPA and HCB, was also observed when metalaxyl-M was included, resulting in a stronger effect of AMPA on the bacterial community composition. These interaction-like patterns have, to the best of our knowledge, not yet been reported in the literature, highlighting a previously unrecognised risk to soil microbial communities. However, other pesticide combinations have shown synergistic effects on microbial abundances and functions (Meidl et al. 2024). The fact that AMPA, likely from recent application of glyphosate, interacted with HCB, a persistent yet long-banned pesticide (Stockholm Convention 2001), raises several questions and concerns that need to be verified. For AMPA however, our results indicate that recently applied pesticides can interact with persistent pesticides, “reactivating” them and leading to interactive effects on the soil microbiome. Possible explanations could include the competition for binding sites, as was observed for other pesticides (Munira & Farenhorst 2017), leading to changes in the bioavailability of the pesticides and increased contact with microorganisms. However, these possible interactions need to be studied in more detail yet highlight the complex interplays multiple pesticides could have. Especially, to draw mechanistic conclusions on those possible interactions, experimental validation is needed.

### Limitations of this study

It is important to mention that the study was not a controlled pesticide/no pesticide setup but used a wide variety of different cropping systems and two different types of management, where one includes the application of synthetic pesticides, and the other prohibits their use. This makes our results representative in real-world scenarios, but also introduces several confounding factors that make it difficult to attribute observed effects to specific pesticide residues. We saw that studies on a smaller scale found management effects (Harkes et al. 2019; Hartman et al. 2018), but in larger scale and longer time spans, the effects are likely overshadowed by abiotic factors. Additionally, depending on the crop type, two different sampling depths were used; this probably affects pesticide distribution in the soils compared to each other, which could have an impact on the results across all CSSs (Table 2). We identified a comparably low number of fungal taxa and abundance; this is a recognised limitation of shotgun metagenomics due to sequencing depth, read length, and the limited availability of fungal reference genomes. The fungal:bacterial ratios observed in the present study are roughly comparable to those reported in previous shotgun metagenomic studies (Fierer et al. 2012; Eisenhofer et al. 2026).

## Conclusion

Although European soils differ largely in their site and environmental conditions, which were the main drivers of microbial composition, we could show that pesticide effects exist in the agricultural fields of Europe, independent of confounding effects of site and environmental conditions. While the farming system just played a minor role in the European context. From the observed effects of fungicides and AMPA, we conclude that the identity of the pesticides present plays a major role that determines these effects. There is a link to a set of modes of action that disrupt processes, not only in the intended targets but also in non-target organisms. By affecting these mechanisms, pesticides possibly affect the soil microbiome and may affect the microbial functions in soils. They might further interact with other pesticides, both recently applied or long banned, yet still present. This should be further investigated, and these persistent “legacy” pesticides should not be overlooked in residue analysis and further studies on mixture effects. This work helps to understand the obvious and the hidden truths of pesticides in European soils and provides a basis for detailed analysis of the processes proposed here.

## Supplementary Information

Below is the link to the electronic supplementary material.

ESM 1 (XLSX 242 KB)

ESM 2 (DOCX 809 KB)

## Data Availability

The authors declare that the data supporting the findings of this study are available within the paper and its Supplementary Information files. Should any raw data files be needed in another format they are available from the corresponding author upon reasonable request.
