# Supplementary material for: Beneath the surface: non-target effects of multiple pesticides on the soil microbiome in organic and conventional agricultural European fields
Source: Environ Sci Pollut Res Int. 2026 Aug 13;33(26):13432–48. doi: 10.1007/s11356-026-38127-7 (PMC13503437; doi:10.1007/s11356-026-38127-7)
Supplement: Supplementary file 1 [file 11356_2026_38127_MOESM2_ESM.docx]

Supplementarty material:

**DNA extraction protocol SPRINT soil samples:**

Adapted from Harkes et al. (2025)

| Step | Procedure |
| --- | --- |
|  |  |
| 1. | Weigh 1 g of thoroughly mixed soil, transfer it to a 15 mL bead tube, add 1.5 g of silicon carbide powder (46 grit) |
| 2. | Add 3 mL of bead solution (181 mM disodium phosphate, 121 mM guanidinium thiocyanate), 0.24 mL of lysis buffer (150 mM NaCl, 4% (w/v) SDS, 0.5 M Tris), |
| 3. | Bead beat the tubes for 15 minutes in a pain shaker or a vortex. |
| 4. | Centrifuge the tubes for 5 minutes (2,500 x g) to separate the soil particles from the lysate. |
| 5. | Transfer 2 mL of the upper aqueous phase to a new 15 mL tube, and add 1 mL of 3 M ammonium acetate. Shake 1 minute by hand to mix. |
| 6. | Centrifuge the tubes for 5 minutes (2,500 x g) to separate the precipitate from the nucleic acids. |
| 7. | Transfer 2.6 mL of the upper aqueous phase to a new 15 mL tube, and add 0.8 mL of 120 mM ammonium aluminum sulfate dodecahydrate solution. Shake 1 minute by hand to mix. |
| 8. | Centrifuge the tubes for 5 minutes (2,500 x g) to separate the precipitate from the nucleic acids. |
| 9. | Avoiding the pellet, transfer 2 mL of supernatant into a clean 15 mL tube |
| 10. | Add 3.2 mL binding solution (at RT) to the supernatant (binding solution: an aqueous solution of 5 M guanidine hydrochloride (GuHCl) and 30 mM Tris-HCl (pH: 6.5) with 9% (v/v) isopropanol) and shake by hand to mix. |
| 11. | The following steps use “Biocomma columns” to bind DNA (column + 2 mL collection tube) and DNase/RNase free tips. |
| 12. | Load 855 µL from step 10 onto each of two “Biocomma column” to bind DNA and spin 10.000 x g 15 sec. Decant supernatant. Repeat this two times until all from step 10 is split between the two spin columns. |
| 13. | Add 500 µL washing buffer ((10mM Tris-HCl, pH: 6.5), 100 mM NaCl, and absolute EtOH final v/v 60%) followed by spinning 10.000 x g 15 seconds. Decant supernatant to avoid overflow. Repeat this step 3x. |
| 14. | Dry spin once more 10.000 x g 15 seconds. |
| 15. | Transfer the columns to a new 1.5 mL collection tube and air dry the column for 8 minutes to remove leftover ethanol. |
| 16. | Add 0.1 mL of elution buffer (10 mM Tris-HCl, pH: 8.0), let soak in for 2 minutes. |
| 17. | Spin first column 10.000 x g 15 seconds. Discard spin column. |
| 18. | Transfer second spin column to same collection tube and spin once more 10.000 x g 15 seconds. |
| 19. | Store collected eluate (0.2 mL) at -20 °C until further use. |

**Parameters for fitting of linear models:**

Linear models with following variables SOC, pH, clay, Water content in per cent, AMPA, and the summed up concentrations of the different pesticide classes, Organochlorines, no data, Organophosphates, Amide, Strobilurin, Triazole, Pyrethroids, Anilide, Benzamide, Morpholine, Aryl phenol ketones, Diamides, Cyclodiene, Dinitroaniline, Carbamates, Neonicotinoids, Pyrazole, Oxadiazines, Nitrophenol ether, Thiocarbamate, Triazinone, Spinosyns, Triazolopyrimidine, Triazine, Urea, Anilineopyrimidine, Phenylpyrrole, Diacylhydrazines, Tetronic and Tetramic acid derivatives, Butenolides, Phenoxypropionic, Anilinopyrimidine, Triazolone, Fungicide, Insecticide, Herbicide and total Copper, as parameters were fitted. When one of the sum parameters appeared in the linear model, it was replaced by the actual parameters to see if the effect was cumulative or from singular pesticides in the group.

**Linear models:**

Method:

step(lm(biovar ~ 1, data = subset(samples_df_EU, Country == country)),

direction = "forward",

scope = ~SOC + pH + clay + Water.content.percent + AMPA + Organochlorines + No.data +	Organophosphates + Amide + Strobilurin + Triazole + Pyrethroids + Anilide + Benzamide+	Morpholine + Aryl.phenol.ketone + Diamides + Cyclodiene + Dinitroaniline + Carbamates+	Neonicotinoids + Pyrazole + Oxadiazines + Nitrophenol.ether + Thiocarbamate + Triazinone +	Spinosyns + Triazolopyrimidine + Triazine + Urea + Anilineopyrimidine + Phenylpyrrole +	Diacylhydrazines + Tetronic.and.Tetramic.acid.derivatives + Butenolides + Phenoxypropionic +	Anilinopyrimidine + Triazolone + Fungicide + Insecticid + Herbicide + Cu..tot.,

data = country_data, trace = TRUE)

Figure S1: Scheme of the statistical workflow and desicion making

Figure S 2 Microbial composition in a) conventional and b) organic fields by country on class level. (CSS Switzerland (CH), CSS the Czech Republic (CZ), CSS Denmark (DK), CSS France (FR), CSS Croatia (HR), CSS Italy (IT), CSS the Netherlands (NL), CSS Portugal (PT), CSS Slovenia (SL) and CSS Spain (ES).

Figure S 3 Microbial composition in a) conventional and b) organic fields by country on phylum level. (CSS Switzerland (CH), CSS the Czech Republic (CZ), CSS Denmark (DK), CSS France (FR), CSS Croatia (HR), CSS Italy (IT), CSS the Netherlands (NL), CSS Portugal (PT), CSS Slovenia (SL) and CSS Spain (ES).

Figure S 4 RDA with the country as the explanatory variable. (CSS Switzerland (CH), CSS the Czech Republic (CZ), CSS Denmark (DK), CSS France (FR), CSS Croatia (HR), CSS Italy (IT), CSS the Netherlands (NL), CSS Portugal (PT), CSS Slovenia (SL) and CSS Spain (ES).

Figure S 5 RDAs for Crop (a) and Country (b) using just the data from CSS_IT, CSS_FR, CSS_ES and CSS_PT

Figure S 6 RDAs on the bacterial community per country, with farming system as explanatory variable. (CSS Switzerland (CH), CSS the Czech Republic (CZ), CSS Denmark (DK), CSS France (FR), CSS Croatia (HR), CSS Italy (IT), CSS the Netherlands (NL), CSS Portugal (PT), CSS Slovenia (SL) and CSS Spain (ES).

Figure S 7 RDAs with the number of pesticides as an explanatory variable in all countries. (CSS Switzerland (CH), CSS the Czech Republic (CZ), CSS Denmark (DK), CSS France (FR), CSS Croatia (HR), CSS Italy (IT), CSS the Netherlands (NL), CSS Portugal (PT), CSS Slovenia (SL) and CSS Spain (ES).

Figure S 8 RDAs with the number of pesticides as an explanatory variable, corrected for the effect of the farming system, in all countries. (CSS Switzerland (CH), CSS the Czech Republic (CZ), CSS Denmark (DK), CSS France (FR), CSS Croatia (HR), CSS Italy (IT), CSS the Netherlands (NL), CSS Portugal (PT), CSS Slovenia (SL) and CSS Spain (ES).

Figure S 8 Selected biological parameters plotted against the number of pesticides detected.

Figure S 9 Measured PLFA concentrations and enzyme activity plotted against the log sum of detected pesticides.

**References:**

Harkes P, van den Elsen S, Helder H, 2025. Microbial DNA isolation from 1 gram soil v1. https://doi.org/10.17504/protocols.io.4r3l29b9jv1y/v1
